# Single-electron detection utilizing coupled nonlinear microresonators

**DOI:** 10.1038/s41378-020-00192-4

**Published:** 2020-10-05

**Authors:** Xuefeng Wang, Xueyong Wei, Dong Pu, Ronghua Huan

**Affiliations:** 1 Department of Mechanics, Key Laboratory of Soft Machines and Smart Devices of Zhejiang Province, Zhejiang University, Hangzhou, 310027 People’s Republic of China; 2 State Key Laboratory for Manufacturing Systems Engineering, Xi’an Jiaotong University, Xi’an, 710049 People’s Republic of China

**Keywords:** Electrical and electronic engineering, Sensors

## Abstract

Since the discovery of the electron, the accurate detection of electrical charges has been a dream of the scientific community. Owing to some remarkable advantages, micro/nanoelectromechanical system-based resonators have been used to design electrometers with excellent sensitivity and resolution. Here, we demonstrate a novel ultrasensitive charge detection method utilizing nonlinear coupling in two micromechanical resonators. We achieve single-electron charge detection with a high resolution up to 0.197 ± 0.056 ${\mathrm{e}}/\sqrt {{\mathrm{Hz}}}$ at room temperature. Our findings provide a simple strategy for measuring electron charges with extreme accuracy.

## Detectors: creating extremely accurate electrometers with ultrahigh resolution

Scientists from China have developed a novel method for significantly enhancing the resolution of electrometers, paving the way for new devices for various applications, including mass spectrometry, surface charge analysis, and aerosol science. Owing to their low cost, high accuracy, and ability to be batch manufactured, microelectromechanical systems (MEMS)-based resonators have been used to design electrometers with excellent sensitivity and resolution. However, their complicated mechanical structure, low environmental temperature, and inability to detect in real-time have limited their applications. A team of Chinese researchers led by Ronghua Huan from Zhejiang University and Xueyong Wei from Xi’an Jiaotong University demonstrated an electrometer that uses two coupled nonlinear MEMS resonators, significantly enhancing its charge detection resolution and sensitivity. The electrometer is capable of measuring single-electron charges extremely accurately at room temperature and with ultrahigh resolution in real-time.

## Introduction

Highly sensitive electrometers have been a focus of research for more than a century and can be used in many diverse applications, such as mass spectrometry^1^, surface charge analysis^2^, particle detection of nuclear studies^3^, and various applications of aerosol science^4^. owing to their low cost, fast response, high accuracy, and ability to be manufactured in batches^5^, micro/nanoelectromechanical system (M/NEMS)-based resonators have been used to design electrometers with excellent sensitivity and resolution^6^. To date, researchers have developed various high-resolution charge sensors utilizing self-assembled quantum dots^7^, MEMS vibrating reeds^8^, carbon nanotubes^9^, mode localization^10^, etc. However, some limits, including the requisite ultralow environmental temperature^6,7,9,11^, a complicated mechanical structure^8^, the required linear dynamic response^6,10,12^ or unachievable real-time detection^6–11,13^, will inevitably hinder their practical application. owing to the size effect^14^, microresonators are more easily excited into the nonlinear regime^15^. Coupling between individual resonators can also lead to complex nonlinear behavior^16,17^. The exploitation of nonlinear phenomena^18–21^ to improve performance has recently attracted significant attention, including mass sensing in terms of coupled nonlinear MEMS resonators^22^ and novel signal amplification schemes through a bifurcation topology^23^. Clearly, bifurcation exists widely in nonlinear systems^24,25^, and it is worthwhile to excavate the potential of nonlinear phenomena for practical applications. The use of multiple resonators also has the advantage of improving common-mode rejection capabilities^26^. Here, we show that the internal resonance of two coupled nonlinear microresonators can significantly enhance the resolution of an electrometer. Ultrasensitive charge detection with a resolution of 0.197 ± 0.056 ${\mathrm{e}}/\sqrt {{\mathrm{Hz}}}$ at room temperature is achieved. The proposed device has a simple structure and can provide real-time measurements.

## Materials and methods

### Fabrication of the microresonator

**Fig. 1:**
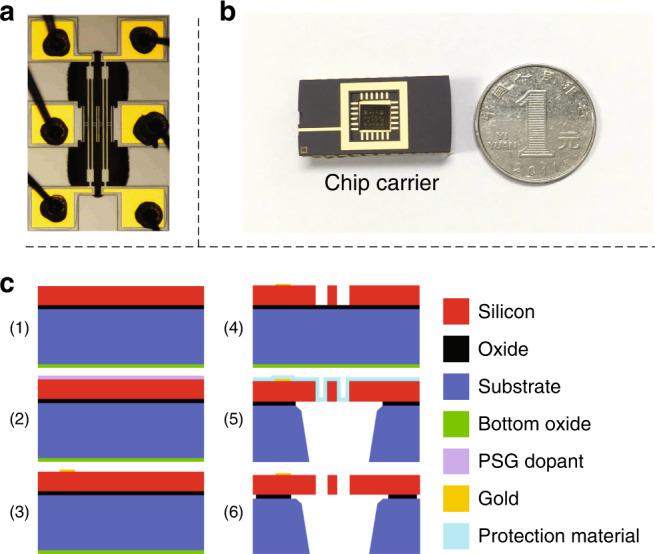
Microresonators and their fabrication. **a** Microscopic image of the microresonators. **b** Comparison of a chip and a coin. **c** SOI-MUMPs micromachining process. The process begins with 25 μm n-type double-sided polished SOI wafers (c1). Then, the top surface of the silicon layer is doped by depositing a phosphosilicate glass (PSG) layer (c2). After annealing, the PSG layer is removed via wet chemical etching. The first deposited layer is a 500 nm gold pad (c3), placed through a liftoff process. Next, silicon is lithographically patterned and etched using deep reactive ion etching (c4). With a frontside protection material on the top surface of the silicon layer, the wafers are reversed to etch the substrate layer from the bottom (c5). The frontside protection material is finally stripped using a dry etching process. The remaining “exposed” oxide layer is removed from the top surface using a vapor hydrogen fluoride process (c6)

The basic structure of the electrometer proposed here consists of two identical silicon-based microresonators, as shown in Fig. 1a. Each microresonator is designed as a widely used structure called a double-ended tuning fork that is 350 μm long, 20 μm wide, and 25 μm thick. Specific sizes can be found in Supplement Material S1. A silicon-based chip is attached to a chip carrier. Figure 1b shows a comparison of a chip and a coin. The microresonators are fabricated through a commercial silicon-on-insulator (SOI)–MUMPs micromachining process^27^, as shown in Fig. 1c.

### Measurement scheme

All measurements are tested in a vacuum chamber at a pressure below 3 Pa at room temperature. The experimental environment is shown in Supplement Material S2.

**Fig. 2:**
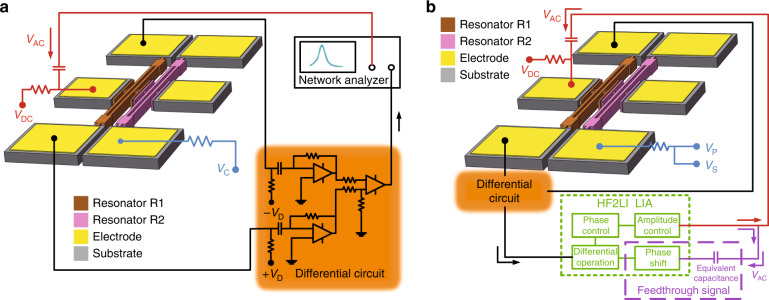
Schematic drawing of the measurement circuit. **a** Open-loop experiment. **b** Closed-loop experiment. The orange part represents the same differential circuit used in the open-loop experiment. The green dotted line block represents some necessary function modules in the HF2LI LIA. The purple dashed line block shows a method to create an equivalent feedthrough signal by utilizing a capacitor and phase shift

The open-loop measurement circuit shown in Fig. 2a is built to characterize this coupled system. As a common detection method, piezoresistive readout^28–30^ is used throughout all the tests because of its unique advantages, such as higher sensitivity and fewer common-mode signal components. An electric voltage $V_{DC} + V_{AC}\,{\mathrm{cos}}({\Omega} \tilde t)$ is loaded to drive resonator R1 to vibrate. DC voltage ±*V*_*D*_ is applied to both sides of actuated resonator R1 to generate a motional current caused by a time-varying electrical resistance due to the piezoresistive effect. Thus, the transverse displacement of the resonant beam can be detected clearly. A network analyzer (Agilent E5061B) performs the frequency sweep operation by outputting sinusoidal motivation and collecting the vibration amplitude and phase. DC voltage *V*_*C*_ is loaded to one side of resonator R2, which generates a coupling voltage *V*_*C*_ across the coupling parallel plates. *V*_*C*_ is accurately controlled through a SourceMeter (KEITHLEY 2400) with a high-stability output.

Figure 2b displays a closed-loop circuit based on the phase-locked loop^31^ (PLL) to track the resonant frequency for real-time testing. A PLL keeps the output signal synchronized with a reference input signal in both frequency and phase. More precisely, the PLL is simply a servo system that controls the phase of its output signal in such a way that the phase error between the output phase and the reference phase reduces to a minimum. In this circuit, the transverse displacement of resonator R1 is first extracted by a differential circuit. After the differential operation and phase and amplitude control, the resulting signal is fed back to drive resonator R1 for vibration. The feedback driving force keeps resonator R1 in oscillation by compensating for the energy dissipation. In closed-loop tests, the resonators are electrically actuated, sensed and embedded using a digital locked-in amplifier (LIA, Zurich Instruments HF2LI). The coupling voltage *V*_*C*_ loaded on the body of resonator R2 is a combination of the polarization voltage *V*_*P*_ and dynamic voltage signal *V*_*S*_. Owing to the coupling interaction, the output frequency of R1 will shift as *V*_*C*_ varies, which implies a possible way to perform dynamic voltage signal detection by measuring the change in oscillation frequency of R1.

### Feedthrough cancellation

Capacitive parasitic feedthrough is an impediment that is inherent to all electrically interfaced MEMS resonators^32^. The feedthrough signal always distorts the real amplitude and phase response of a resonator. Hence, the feedthrough signal has to be properly eliminated.

In the open-loop experiment, we collect a response signal mixed with a feedthrough signal (with *V*_*DC*_ on) and a pure feedthrough signal (with *V*_*DC*_ off) through a frequency sweep. The feedthrough signal can be eliminated by using the program codes provided in Supplement Material S7. In the closed-loop tests, a differential operation is used to remove the feedthrough signal in real time.

## Results

### The basic mechanism of high-resolution charge detection

**Fig. 3:**
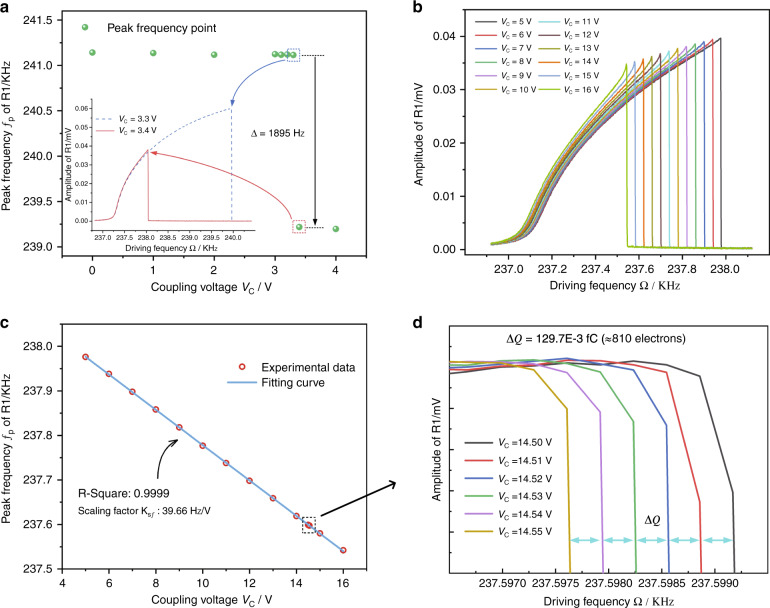
Results of the open-loop experiment. All tests are processed with the same conditions, i.e., *V*_*AC*_ = 316.2 mV and *V*_*DC*_ = 18 V. **a** Peak frequency *f*_*P*_ of the amplitude-frequency curves of R1 for different coupling voltages. The inset shows the amplitude-frequency curves for a coupling voltage below the threshold (blue dashed line) and above the threshold (red solid line). **b** Measured responses of R1 for *V*_*C*_ values ranging from 5 V to 16 V with a 1 V step. **c** Measured peak frequency of R1 shifts vs the coupling voltage. **d** Measured responses of R1 for *V*_*C*_ values ranging from 14.50 V to 14.55 V with a 0.01 V step

In open-loop measurement, we obtain the amplitude-frequency responses of R1 for varying coupling voltage *V*_*C*_. The corresponding peak frequency with increasing *V*_*C*_ is plotted in Fig. 3a as the green dots. An 1895 Hz discontinuous jump of the peak frequency is observed owing to the nonlinear coupling of two resonators when *V*_*C*_ reaches a critical value *V*_*C*_ = 3.4 V. The inset of Fig. 3a shows the amplitude-frequency curves of R1 for *V*_*C*_ below and above the critical value, which further demonstrates this discontinuous phenomenon.

Figure 3b shows the amplitude–frequency curves for uniformly increasing *V*_*C*_ above the critical value. It is shown that the peak frequency of R1 varies continuously with *V*_*C*_ again once *V*_*C*_ crosses the critical value. Figure 3c plots the peak frequency of R1 as a function of *V*_*C*_, which reveals an extremely linear relation between the peak frequency shift and *V*_*C*_ with a fitting *R*^2^ coefficient of 0.9999. Figure 3d shows the response curves of R1 when the coupling voltage varies with a very small step of 0.01 V, where linear variation can still be detected clearly. According to ${\mathrm{{\Delta} }}Q = C{\mathrm{{\Delta} }}V_C$, the equivalent variation in charge Δ*Q* for small voltage step Δ*V*_*C*_ = 0.01 V is calculated as Δ*Q* = 129.7E-3 fC (~810 electrons), where *C* = 0.01297 pF is the capacitance between two sensing electrodes (the fringe effect is considered as shown in Supplement Material S3). The linear dependence of the frequency shift on the coupling voltage provides a high-resolution method for charge detection. This device implements charge detection as follows: the input charges change the coupling voltage *V*_*C*_, which leads to a linear shifting of the peak resonant frequency of R1 that can be tracked in real time by the PLL. Meanwhile, as shown in Fig. 3c, the coupling voltage *V*_*C*_ has a linear range of more than 11 V; thus, the electrometer has a broad charge sensing region of up to ~1000000 electrons according to ${\mathrm{{\Delta} }}Q = C{\mathrm{{\Delta} }}V_C$.

### Numerical simulation

According to the measured response curves, R1 and R2 exhibit strong Duffing-like nonlinearity. Considering the coupling between these two resonators, a coupled Duffing model of the microsystem is formulated as follows^33^:

1 $$\begin{array}{*{20}{l}}m_1\displaystyle\frac{{d^2\tilde x}}{{d\tilde t^2}} + c_1\frac{{d\tilde x}}{{d\tilde t}} + k_1\tilde x + {\Gamma} _1\tilde x^3 = \frac{1}{2}\left| {\frac{\partial }{{\partial \tilde x}}\left( {\frac{{S{\it{\epsilon }}_r}}{{d - \tilde x}}} \right)} \right|\left[ {V_{AC}\,{\mathrm{cos}}\left( {{\Omega} \tilde t} \right) + V_{DC}} \right]^2\\ - \, \displaystyle\frac{1}{2}\left| {\frac{\partial }{{\partial \left( {\tilde x - \tilde y} \right)}}\left( {\frac{{S{\it{\epsilon }}_r}}{{d + \tilde x - \tilde y}}} \right)} \right|V_C^2 m_2\frac{{d^2\tilde y}}{{d\tilde t^2}} + c_2\frac{{d\tilde y}}{{d\tilde t}} + k_2\tilde y + {\Gamma} _2{\tilde{\mathrm y}}^3 = \frac{1}{2}\left| {\frac{\partial }{{\partial \left( {\tilde x - \tilde y} \right)}}\left( {\frac{{S{\it{\epsilon }}_r}}{{d + \tilde x - \tilde y}}} \right)} \right|V_C^2\end{array}$$

where *m*_1_ and *m*_2_ are the equivalent masses of R1 and R2, respectively; $\tilde x$ and $\tilde y$ are the equivalent transverse displacements of R1 and R2, respectively; *c*_1_ and *c*_2_ are the equivalent viscous damping coefficients; *k*_1_ and *k*_2_ are the equivalent linear stiffness coefficients; Γ_1_ and Γ_2_ are the equivalent cubic nonlinear stiffness coefficients; *S* is the area of the driving or coupling electrode; ${\it{\epsilon }}_r$ is the dielectric constant; *d* is the initial spacing between electrodes; *V*_*DC*_ and *V*_*C*_ are the DC polarization voltages for driving and coupling, respectively; and *V*_*AC*_ and Ω are, respectively, the amplitude and frequency of the AC driving voltage.

Utilizing Taylor series expansion and ignoring high-order coupling terms, the nondimensional form of Eq. (1) is obtained^26^:

2 $$\begin{array}{*{20}{l}}\displaystyle\frac{{d^2x}}{{dt^2}} + Q^{ - 1}\frac{{dx}}{{dt}} + x + \gamma x^3 = f{\mathrm{cos}}\left( {\omega t} \right) + \alpha \left( {x - y} \right)\\ \displaystyle\frac{{d^2y}}{{dt^2}} + Q^{ - 1}\frac{{dy}}{{dt}} + p^2y + \gamma y^3 = \alpha \left( {y - x} \right)\end{array}$$

where $x = \frac{{\tilde x}}{d}$ and $y = \frac{{\tilde y}}{d}$ are the nondimensional displacements; $\omega _1 = \sqrt {k_1/m_1}$ and $\omega _2 = \sqrt {k_2/m_2}$ are the natural resonant frequencies, with $\omega _1 \approx \omega _2$; $p = \omega _2/\omega _1 > 1$; $t = \omega _1\tilde t$; $Q = \frac{{c_1}}{{m_1\omega _1}} \approx \frac{{c_2}}{{m_2\omega _1}}$ is the quality factor; $\gamma = \frac{{{\Gamma} _1d^2}}{{m_1\omega _1^2}} \approx \frac{{{\Gamma} _2d^2}}{{m_2\omega _1^2}}$ is the normalized cubic nonlinear stiffness coefficient; $f = \frac{{S{\it{\epsilon }}_rV_{AC}V_{DC}}}{{m_1\omega _1^2d^3}}$ is the normalized amplitude of the electrostatic excitation; $\omega = \frac{{\Omega} }{{\omega _1}}$ is the normalized electrostatic excitation frequency; and $\alpha = \frac{{S{\it{\epsilon }}_rV_C^2}}{{m_1\omega _1^2d^3}} \approx \frac{{S{\it{\epsilon }}_rV_C^2}}{{m_2\omega _1^2d^3}}$ is the strength of the electrostatic coupling.

**Fig. 4:**
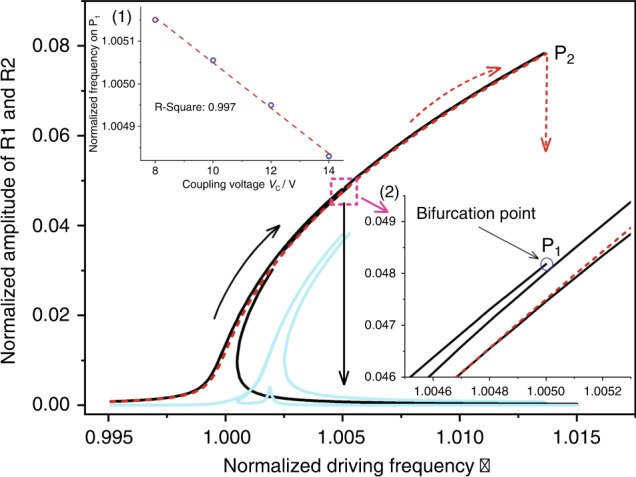
Numerical amplitude–frequency curves of both resonators. The dashed red line represents the response of R1 for coupling voltages below the critical value. The solid black and cyan lines represent the responses of R1 and R2, respectively, for coupling voltages above the critical value. Arrows denote the trend of the responses. Inset (1) shows that the frequency at the bifurcation point P_1_ decreases linearly as the coupling voltage increases. The circles represent the results from the numerical simulation. The red dotted line is a fitting curve. Inset (2) is an enlarged image of the dashed box. It clearly shows the existence of a bifurcation point in this coupled system when the coupling voltage reaches the threshold

Generally, Eq. (2) cannot be solved analytically owing to its complicated solution branches. Thus, numerical simulations of the amplitude–frequency curves of this system are obtained and plotted in Fig. 4 through a numerical method called the time-domain collocation method^34^ with parameters extracted from the experiments (details of the procedure are displayed in Supplement Material S5). A bifurcation point P_1_ is observed when the coupling voltage *V*_*C*_ reaches a threshold, which can exactly explain the discontinuous jump phenomenon of the peak frequency in Fig. 3a. The bifurcation point is owing to the internal resonance between these two resonators. With increasing coupling strength, more energy is transferred from actuated R1 to coupled R2. When the coupling voltage reaches a threshold, R1 does not have enough energy to maintain a large amplitude, thus causing the amplitude of R1 to drop to the lower branch, which leads to the discontinuous phenomenon. The inset (1) of Fig. 4 shows that the frequency at the bifurcation point decreases linearly as the coupling voltage increases with a fitting *R*^2^ coefficient of 0.997, which agrees with the experimental results in Fig. 3c.

In the above studies, the capacitance *C* between electrodes is assumed to be constant. However, owing to the relative motion of the two sensing electrodes, the capacitance *C* may vibrate. The effect of the vibration of *C* on charge detection has been investigated analytically and proved to be negligible (see Supplement Material S4).

### Real-time charge detection method

**Fig. 5:**
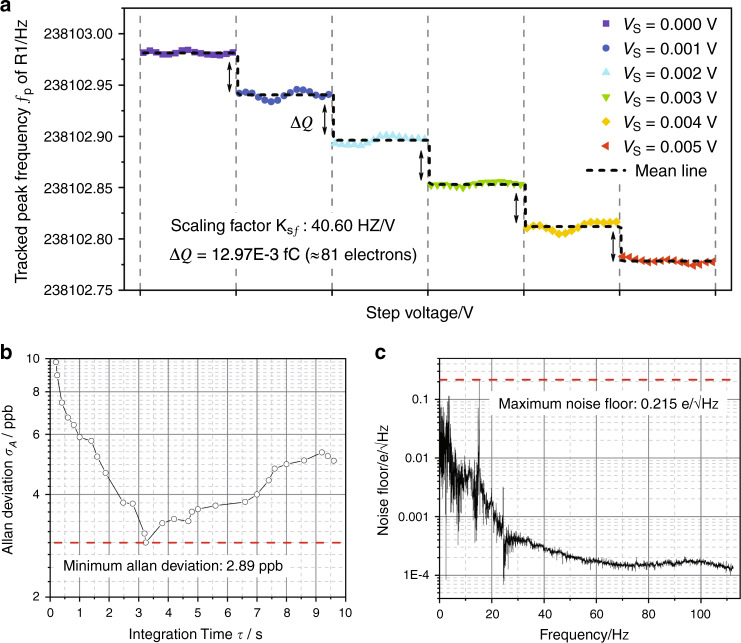
Results of the closed-loop experiment. All tests are processed with the same conditions, i.e., *V*_*AC*_ = 316.2 mV and *V*_*DC*_ = 18 V. **a** Detected step response of R1 for a varying coupling voltage. The symbols represent the experimental data of the response frequency from the LIA, and the solid line denotes the mean value in one test. **b** Allan deviation of oscillator R1. **c** Measured noise floor of oscillator R1 in one test

A closed-loop circuit is set up for real-time charge detection, utilizing the above linear dependence of the peak frequency on the coupling voltage, as shown in Fig. 2b. Here, coupling voltage *V*_*C*_ is a combination of polarization voltage *V*_*P*_ and dynamic voltage signal *V*_*S*_. *V*_*P*_ is a fixed voltage that is set above the critical value as *V*_*P*_ = 4 V (>3.4 V) to ensure that the resonators work in the linear region, whereas *V*_*S*_ is a step voltage signal used to imitate the changing voltage caused by external charge. In Fig. 5a, we plot the step response when the coupling voltage *V*_*C*_ varies with a tiny step 0.001 V (equivalent variation in charge is 12.97E-3 fC). The variation in the peak frequency of R1 is clearly identified, which indicates a much higher (10-fold) resolution for charge variation than in the open-loop measurement. The time-domain response for the step voltage signal can be seen in Supplement Material S6.

The resolutions of charge detection are normally calculated in two different ways by using the Allan deviation or noise floor. To obtain the Allan deviation and noise floor, we need to track a time series of the peak frequency of the amplitude-frequency curves of R1. The frequency fluctuation data are recorded at a fixed sample time of 1/225 s for a duration of 300 s using an LIA-based PLL.

The Allan deviation *σ*_*A*_ is a common indicator to evaluate the frequency stability^35^, which is given by the frequency fluctuations averaged over an integration time *τ* and can be expressed as^21^

3 $$\sigma _A(\tau ) = \sqrt {\frac{1}{{2(N - 1)}}\mathop {\sum}\limits_{i = 1}^{N - 1} {\left( {\overline {f_p} _{i + 1}^\tau - \overline {f_p} _i^\tau } \right)^2} }$$

where $\overline {f_p} _i$ are the relative frequency fluctuations averaged over the ith discrete integration of *τ*. Figure 5b plots the Allan deviation *σ*_*A*_ of the tracked peak frequency of R1 of one test as a function of the integration time *τ*, from which the minimum Allan deviation is observed to be 2.89 ppb. Then, the resolution *R* of charge detection is calculated to be 2.19E-4 fC according to $R = C \cdot \delta f/K_{sf}$ ($\delta f$ is the frequency fluctuation of resonator R1, which equals the Allan deviation $\sigma _A$ multiplied by the characteristic frequency (i.e., *f*_*p*_); *K*_*sf*_ represents the scaling factor of the peak frequency shift to coupling voltage *V*_*C*_, which is 40.60 Hz/V from Fig. 5a), which is equivalent to the charge provided by ~1.3 electrons. To pursue a more credible presentation with a statistical property, multiple independent tests were completed with a mean value of 4.66 ppb and a standard deviation of 1.96 ppb of the minimum Allan deviation. Thus, the resolution *R* is calculated to be 3.53E-4 ± 1.45E-4 fC, which equals the charge provided by ~2.1 ± 0.9 electrons.

The noise floor here is experimentally achieved by fast Fourier transformation analysis of the time series data of the recorded frequency^36^. Figure 5c is the noise floor of the peak frequency of one test with a maximum value of 0.215 ${\mathrm{e}}/\sqrt {{\mathrm{Hz}}}$. Similarly, multi-independent tests are completed, and a mean value of 0.197 ${\mathrm{e}}/\sqrt {{\mathrm{Hz}}}$ and standard deviation of 0.056 ${\mathrm{e}}/\sqrt {{\mathrm{Hz}}}$ of the maximum noise floor are obtained. Therefore, a resolution of 0.197 ± 0.056 ${\mathrm{e}}/\sqrt {{\mathrm{Hz}}}$ is obtained. Single-electron charge detection at room temperature is thus achievable using this device.

## Conclusions

In summary, we presented a new charge detection method with ultrahigh resolution utilizing two coupled nonlinear MEMS resonators. This kind of detection concept has not been reported in previous studies. Beyond unfavorable effects, nonlinearity can greatly enhance the resolution of an electrometer, which sheds light on the considerable potential of nonlinear applications. We explored the complex dynamic response of the aforementioned system through a numerical method. These numerical results demonstrated the emergence of the discontinuous phenomenon in this system and further showed the linear relation between the peak frequency shift and the coupling voltage. We also built a real-time closed-loop measurement circuit for charge detection.

## Supplementary information

Supplementary materials
