# Supplementary material for: Single-electron detection utilizing coupled nonlinear microresonators
Source: Microsyst Nanoeng. 2020 Oct 5;6:78. doi: 10.1038/s41378-020-00192-4 (PMC8433136; doi:10.1038/s41378-020-00192-4)
Supplement: Supplementary file 1 — Supplementary materials [file 41378_2020_192_MOESM1_ESM.docx]

Supplementary Materials for

Single-electron detection utilizing coupled nonlinear micro-resonators

Xuefeng Wang^1^, Xueyong Wei^2^, Dong Pu^1^, Ronghua Huan^1^

1 Department of Mechanics, Key Laboratory of Soft Machines and Smart Devices of Zhejiang Province, Zhejiang University, Hangzhou 310027, People’s Republic of China

2 State Key Laboratory for Manufacturing Systems Engineering, Xi’an Jiaotong University, Xi’an 710049, People’s Republic of China

S1. Structure of the Resonator

Fig. S1 Schematic view of the micro-resonator.

Table S1 Some dimensions of the charge sensor

S2. Experimental Setup

Fig. S2 The experimental setup and some relative circuits.

S3. The Influence of Fringe Effect

S4. Feasibility Analysis

Fig. S3 The equivalent mass-spring-damper model of the micro-resonator.

S5. Time-domain Collocation Method

S6. Original Experimental Data

Fig. S4 The time domain response for step voltage signal

S7. Feedthrough Signal Elimination Code

***S1. Structure of the Resonator***

Fig.S1 shows the schematic view of the micro-resonator. The micro-resonator is designed as widely used structure called double-ended tuning fork for vast advantages, such as minimized energy dissipation and common-mode rejection of acceleration. The endings of the tuning fork are anchored in gold electrodes which are connected with external electrical sources. The outboard plates near gold electrodes are utilized for electrostatic actuation. The plates between two tuning forks are used to produce electrostatic coupling. A summary of the dimensions and design parameters assigned for the charge sensor is given in Table S1.


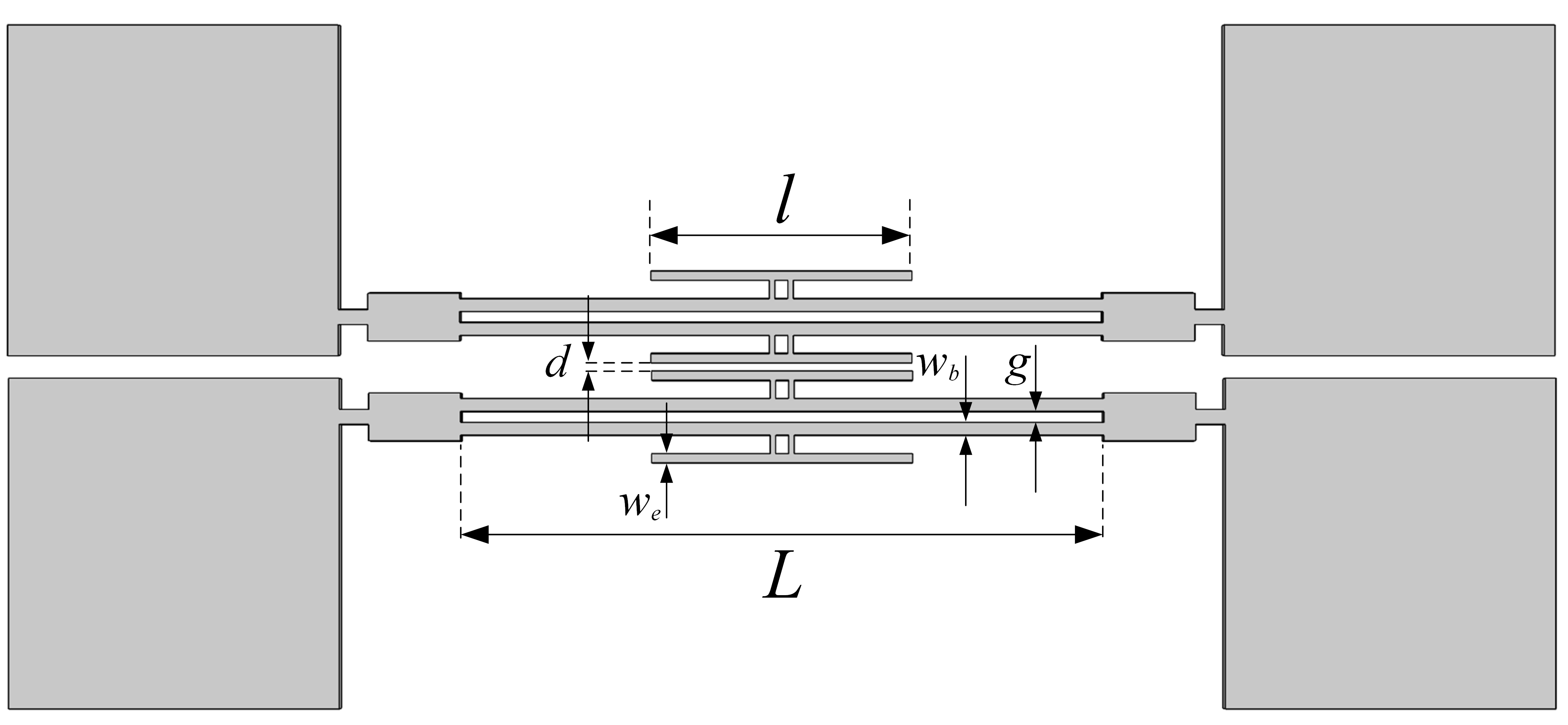


**Fig.S1.** **Schematic view of the micro-resonator.**

| **Table S1. Some dimensions of the charge sensor** | |
| --- | --- |
| **Parameters** | **Dimensions /** $\mu m$ |
| Device thickness ***h*** | 25 |
| Beam length ***L*** | 350 |
| Beam width ***w_b_*** | 7 |
| Electrode length ***l*** | 142 |
| Electrode width ***w_e_*** | 5 |
| Gap between beams ***g*** | 6 |
| Gap between resonators ***d*** | 3 |

***S2. Experimental Setup***

***
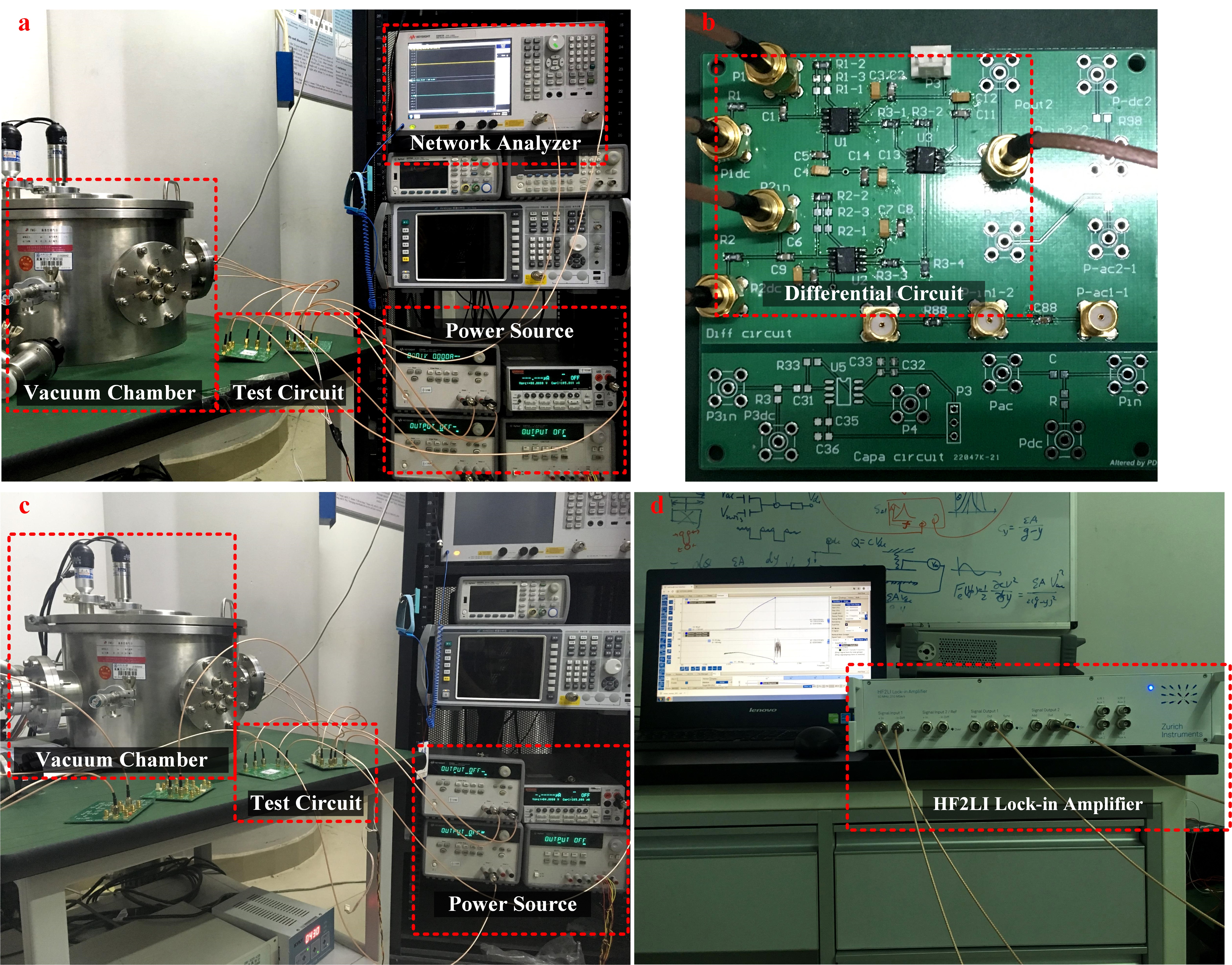
***

**Fig.S2.** **The experimental setup and some relative circuits**. **a.** The setup of open-loop experiment. **b.** The differential circuit in open-loop experiment. **c.** The setup of closed-loop experiment. **d.** The HF2LI Lock-in Amplifier in closed-loop experiment.

***S3. The Influence of Fringe Effect***

An ideal parallel plate capacitance can be expressed by

$C_{0}=\frac{\epsilon_{r}hl}{d}$ (s1)

where $\epsilon_{r}$ is the permittivity.

Consider the fringe effect, a modified capacitance-computing equation is presented according to a reference 1:

$C=\frac{\epsilon_{r}hl}{d}+\frac{\epsilon_{r}h}{\pi}\left\{ 1+ln\left[ 1+\frac{2\pi l}{d}+ln\left( 1+\frac{2\pi l}{d} \right) \right] \right\}+\frac{\epsilon_{r}l}{\pi}\left\{ 1+ln\left[ 1+\frac{2\pi h}{d}+ln\left( 1+\frac{2\pi h}{d} \right) \right] \right\}$ (s2)

With the relative parameters as shown in Table S1, the ideal capacitance $C_{0}$ can be calculated as 0.01047 pF. However, the modified capacitance is 0.01297 pF.

***S4. Feasibility Analysis***

As shown in Fig.2, the approximated dynamic model of this system can be simplified as a mass-spring-damper system with a linearly coupling term^2^

$m_{1}\ddot{x}+c_{1}\dot{x}+k_{1}x+\Gamma_{1}x^{3}=Fcos\left( \Omega t \right)+J_{c}\left( x-y \right)$

$m_{2}\ddot{y}+c_{2}\dot{y}+k_{2}y+\Gamma_{2}y^{3}=J_{c}\left( y-x \right)$ (s3)

where $m_{1}$, $m_{2}$ are the equivalent mass of R1 and R2, respectively; $x$, $y$ are the equivalent transverse displacements of R1 and R2, respectively; $c_{1}$, $c_{2}$ are the equivalent viscous damping coefficients; $k_{1}$, $k_{2}$ are the equivalent linear stiffness coefficients; $\Gamma_{1}$, $\Gamma_{2}$ are the the equivalent cubic nonlinear stiffness coefficients $F$ is the amplitude of the electrostatic excitation; $\Omega$ is the electrostatic excitation frequency; $J_{c}$ is the strength of electrostatic coupling.


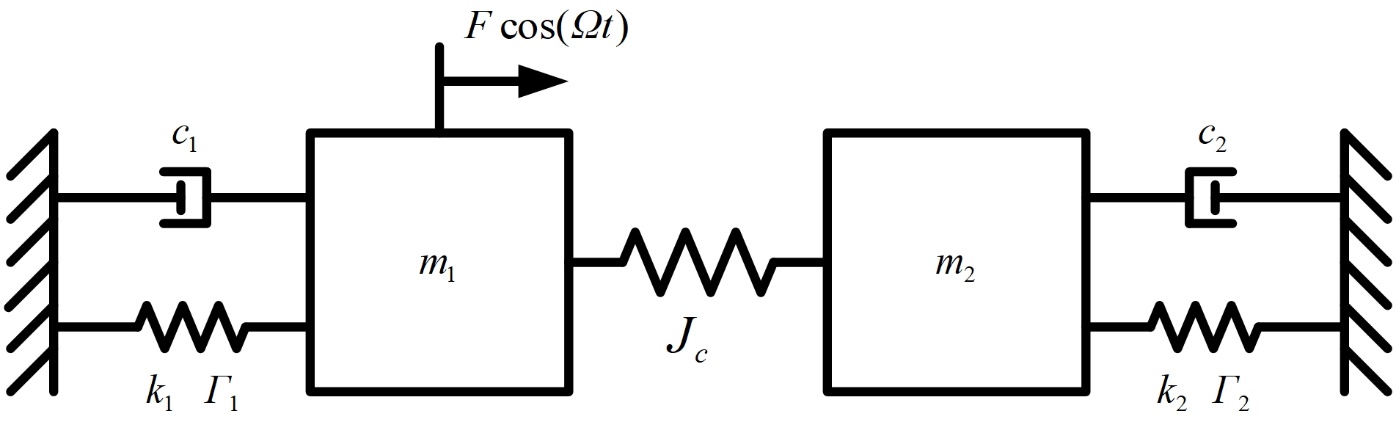


**Fig.S3. The equivalent mass-spring-damper model of the micro-resonator.**

Thus, the transverse deflection of the electrodes for both resonators can be assumed to satisfy the following equations^3^:

$\overset{̂}{x}=acos(\omega t+\phi_{1})$, $\overset{̂}{y}=bcos(\omega t+\phi_{2})$ (s4)

Meanwhile, to simplify the analysis, it is reasonable to consider $a\approx b$ according to the vibration theory. Then, the following equation is derived

$\overset{̂}{x}-\overset{̂}{y}=-2asin(\frac{\phi_{1}}{2}-\frac{\phi_{2}}{2})sin(\omega t+\frac{\phi_{1}}{2}+\frac{\phi_{2}}{2})$ (s5)

Without generality loss, when $\phi_{1}+\phi_{2}=-\pi$, Eq.(s5) can be simplified as

$\overset{̂}{x}-\overset{̂}{y}\leq-2acos(\omega t)\triangleq Acos(\omega t)$ (s6)

Ignoring fringe effect, the continuously changing capacitance $C_{0}$ can be rewritten as

$C_{0}(t)=\frac{hl\epsilon_{r}}{d-\overset{̂}{x}+\overset{̂}{y}}\approx\frac{hl\epsilon_{r}}{d+Acos(\omega t)}$ (s7)

The averaged charge $\overline{Q}$ in a period can be integrated as

$\overline{Q}=\frac{1}{T}\int_{0}^{T}C_{0}(t)Vdt=\frac{1}{T}\int_{0}^{T}\frac{\epsilon_{r}hlV}{d+Acos(\omega t)}dt=\frac{\epsilon_{r}hlV}{\sqrt{d^{2}-A^{2}}}$ (s8)

where $T=\frac{2\pi}{\omega}$, $V$ is the voltage between two electrodes.

Consider the limit case that resonators' vibrations approach pull-in induced failures (i.e. $A=d/3$), $\overline{Q}=1.061Q$, where $Q$ is static charge.

Considering fringe effect Eq.(s2) and pull-in induced failure, the averaged charge in a period can be integrated as

$$\overline{Q}=\frac{1}{T}\int_{0}^{T}C(t)Vdt=1.050Q$$

These results indicate that the influence of changing $C$ is negligible for $\overline{Q}$. Thus, instead of static charge $Q$, averaged charge $\overline{Q}$ can be used to evaluate the charge resolution.

***S5. Time-domain Collocation Method***

In this section, we introduce the time-domain collocation in detail. For the aforementioned system Eq.(s4), we can obtain a nondimensional form

$$\frac{d^{2}x}{dt^{2}}+Q^{-1}\frac{dx}{dt}+x+\gamma x^{3}=fcos\left( \omega t \right)+\alpha\left( x-y \right)$$

$\frac{d^{2}y}{dt^{2}}+Q^{-1}\frac{dy}{dt}+p^{2}y+\gamma y^{3}=\alpha\left( y-x \right)$ (s9)

The harmonic solution of Eq.(s9) is sought in the form:

$$x=A_{0}+\overset{N}{\underset{n=1}{\sum}}A_{n}cos\left( n\omega t \right)+\overset{N}{\underset{n=1}{\sum}}B_{n}sin\left( n\omega t \right)$$

$y=C_{0}+\overset{N}{\underset{n=1}{\sum}}C_{n}cos\left( n\omega t \right)+\overset{N}{\underset{n=1}{\sum}}D_{n}sin\left( n\omega t \right)$ (s10)

The assumed form of $x$ and $y$ can be simplified by considering the symmetrical property of the nonlinear restoring force. Firstly, when the the order of nonlinearity is odd, $A_{0}$ and $C_{0}$ can be discarded^4^. Secondly, it is demonstrated numerically and theoretically that the even harmonic components in Eq.(s10) are zero^5^. Thus, the approximate solution is simplified to

$$x=\overset{N}{\underset{n=1}{\sum}}A_{n}cos\left( 2n\omega t-\omega t \right)+\overset{N}{\underset{n=1}{\sum}}B_{n}sin\left( 2n\omega t-\omega t \right)$$

$y=\overset{N}{\underset{n=1}{\sum}}C_{n}cos\left( 2n\omega t-\omega t \right)+\overset{N}{\underset{n=1}{\sum}}D_{n}sin\left( 2n\omega t-\omega t \right)$ (s11)

where, $N$ is the number of harmonics.

Using the time-domain allocation method within a period of oscillation, we obtain the residual-error function $R(t)$ by substituting Eq.(s11) into Eq.(s9).

$$R(t)=\ddot{x}+Q^{-1}\dot{x}+x+\gamma x^{3}-fcos\left( \omega t \right)-\alpha\left( x-y \right)\neq0$$

$R(t)=\ddot{y}+Q^{-1}\dot{y}+p^{2}y+\gamma y^{3}-\alpha\left( y-x \right)\neq0$ (s12)

Upon enforcing $R(t)$ to be zero at $4N$ equidistant points $t_{j}$ over one period, we can obtain $4N$ nonlinear algebraic equations:

$R_{j}(A_{1},\cdots A_{n};B_{1},\cdots B_{n};C_{1},\cdots C_{n};D_{1},\cdots D_{n})\triangleq0$ (s13)

Finally, the coefficients in Eq.(s13) can be solved by the Newton-Raphson method. We emphasize that the Jacobian matrix $\mathbf{J}$ of Eq.(s13) can be readily derived upon differentiating $R_{j}$ with respect to $A_{i}$, $B_{i}$, $C_{i}$ and $D_{i}$.

$\mathbf{J}=\left[ \frac{\partial R_{j}}{\partial A_{i}},\frac{\partial R_{j}}{\partial B_{i}},\frac{\partial R_{j}}{\partial C_{i}},\frac{\partial R_{j}}{\partial D_{i}} \right]_{4N\times4N}$ (s14)

***S6. Original Experimental Data***

***
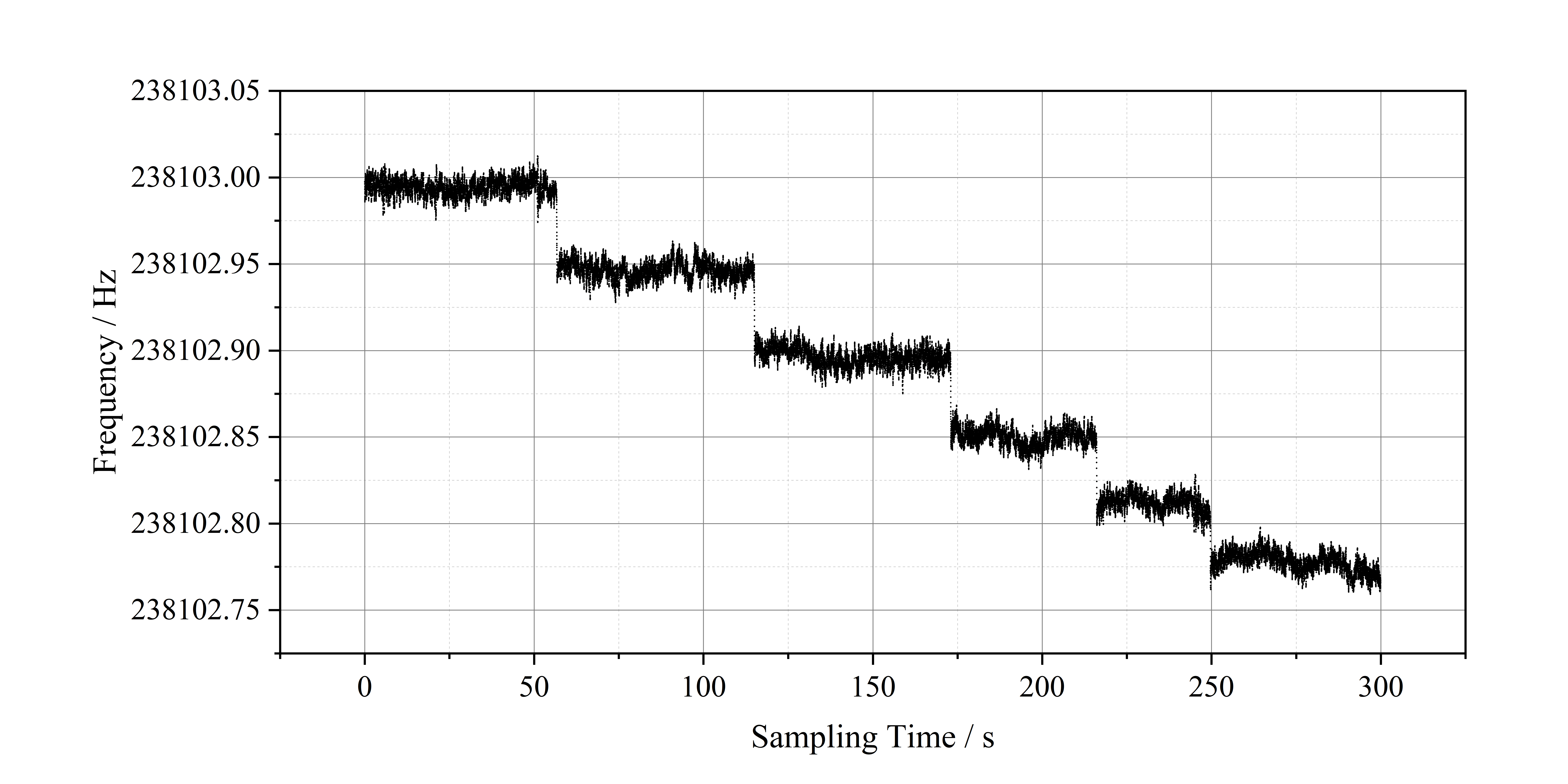
***

**Fig.S4.** **The time domain response for step voltage signal**

***S7. Feedthrough Signal Elimination Code***

clear all;

clc;

%-----crucial!!---average for eliminatefeedthrough--

%-----sp-ep1:first severalpoints-sp-ep2 last points-

sp1=1;

ep1=15;

sp2=1201-15;

ep2=1201;

%$$$$$$$$----------start----------$$$$$$$

%-------Reading s2p results(device on)------

MotionDataFile = ‘ .S2P’; %FileRoutine;

hs1 = sparameters(MotionDataFile);

%data = read(rfdata.data,MotionDataFile);

%-------Reading s2p results(device off)-----

FeedthoughDataFile = ‘ .S2P’; %FileRoutine;

hs2 = sparameters(FeedthoughDataFile);

freq = hs1.Frequencies;

%freq = hs2.Frequencies';

%-------Amp-freq Response (device on)-------

figure(1)

subplot(2,1,1)

ampline = rfplot(hs1,2,1);

subplot(2,1,2)

phaline = rfplot(hs1,2,1,'angle');

title('raw-response');

%-------Amp-freq Response (device off)-------

figure(2);

subplot(2,1,1)

ampline0 = rfplot(hs2,2,1);

subplot(2,1,2)

phaline0 = rfplot(hs2,2,1,'angle');

title('feedthrough');

%-------Contrast on-off Amp response--------

figure(4);

hs1.Frequencies = hs1.Frequencies;

rfplot(hs1,2,1);

hold on;

rfplot(hs2,2,1);

title('before-compensation');

%-----------Eliminating Feedthough---------

AMP_ON=ampline.YData'; %Amplitude device on

PHASE_ON=phaline.YData'; %Phase device on

AMP_OFF=ampline0.YData'; %Amplitude device off

PHASE_OFF=phaline0.YData'; %Phase device off

%--------------compensation----------------

dataa1=AMP_ON(sp1:ep1); %

dataa2=AMP_OFF(sp1:ep1);

avera1=mean(dataa1);

averb1=mean(dataa2);

datab1=AMP_ON(sp2:ep2); %

datab2=AMP_OFF(sp2:ep2);

averb2=mean(datab2);

avera2=mean(datab1);

G1=avera1-averb1;

G2=avera2-averb2;

G=G1/2+G2/2;

AMP_FEED=AMP_OFF+G;

figure;

plot(freq,AMP_ON,'-b',freq,AMP_FEED,'-r');

title('after-compensation');

AMP0_LIN=10.^(AMP_FEED/20);

AMP6_LIN=10.^(AMP_ON/20);

ANG2RAD=(PHASE_ON-PHASE_OFF+90)/180*pi;

A1=AMP6_LIN.*cos(ANG2RAD);

B1=AMP6_LIN.*sin(ANG2RAD);

B3=B1-AMP0_LIN;

AMP_LIN=sqrt(A1.^2+B3.^2);

AMP_dB=20.*log10(AMP_LIN);

PHASE=atan(B3./A1)/pi*180;

figure;

subplot(2,1,1);

plot(freq,AMP_dB,'-b');

subplot(2,1,2);

plot(freq,PHASE,'-r');

title('amp-freq（without-feedthrough）');

***Reference***

1. Dong L, Li Y, Yan H et al. Effects of Electric Field Fringe on Performances of Grid Strip Capacitive MEMS Devices. *High Technology Letters* 2011; **17**: 207-213.
2. Thiruvenkatanathan P, Yan J, Woodhouse J et al. Enhancing parametric sensitivity in electrically coupled MEMS resonators. *Journal of Microelectromechanical Systems* 2009; **18**: 1077-1086.
3. Pandit M, Zhao C, Sobreviela G et al. Utilizing energy localization in weakly coupled nonlinear resonators for sensing applications. *Journal of Microelectromechanical Systems* 2019; **28**: 182-188.
4. Hayashi, C. Subharmonic oscillations in nonlinear systems. *Journal of Applied Physics* 1953; **24**: 521-529.
5. Urabe, M. Numerical investigation of subharmonic solutions to Duffing's equation. *Publications of the Research Institute for Mathematical Sciences* 1969; **5**: 79-112.
